# PERSONALITY AND FAMILY HISTORY OF ALZHEIMER’S DISEASE AS PREDICTORS OF OLDER ADULTS’ SELF-REPORTED MEMORY PROBLEMS

**DOI:** 10.1093/geroni/igz038.806

**Published:** 2019-11-08

**Authors:** Sakshi Bhargava, Nikki Hill, Jacqueline Mogle, Tyler R Bell, Rachel Wion

**Affiliations:** 1 Penn State College of Nursing, University Park, Pennsylvania, United States; 2 The Pennsylvania State University, University Park, Pennsylvania, United States

## Abstract

Understanding individual factors (e.g., personality) associated with self-reported memory problems is important to refine identification of individuals at a higher risk of developing Alzheimer’s disease (AD). Using multilevel modeling, we examined the association of family history of AD and personality traits with self-reported memory problems in older adults (n = 421; 72.21% White; 62.95% female; Mage = 76.69). Results showed that individuals with a family history of AD reported more frequent memory problems and greater one-year memory decline. Similar findings were reported for individuals with higher extraversion scores. Further, older adults with higher neuroticism scores reported greater one- and ten-year memory decline. Neuroticism was positively related to frequency of memory problems, but only among participants with a family history of AD. Findings suggest that higher neuroticism and lower extraversion may increase older adults’ reports of memory problems. Family history of AD may further exacerbate this tendency.

